# What are the recommendations for returning athletes who have experienced long term COVID-19 symptoms?

**DOI:** 10.1080/07853890.2021.1992496

**Published:** 2021-11-02

**Authors:** Rosie K. Lindsay, Jason J. Wilson, Mike Trott, Olawale Olanrewaju, Mark A. Tully, Guillermo F. López-Sánchez, Jae Il Shin, Damiano Pizzol, Peter Allen, Laurie T. Butler, Yvonne Barnett, Lee Smith

**Affiliations:** a Vision and Hearing Sciences Research Centre, Anglia Ruskin University, Cambridge, UK; b Sport and Exercise Sciences Research Institute, School of Sport, Ulster University, UK; c Vision and Eye Research Institute, School of Medicine, Faculty of Health, Education, Medicine and Social Care, Anglia Ruskin University, Cambridge, UK; d The Cambridge Centre for Sport and Exercise Sciences, Anglia Ruskin University, Cambridge, UK; e School of Health Sciences, Institute of Mental Health Sciences, Ulster University, Newtownabbey, UK; f Department of Pediatrics, Yonsei University College of Medicine, Seoul, South Korea; g Italian Agency for Development Cooperation, Khartoum, Sudan ZIP; h Faculty of Science and Engineering, Anglia Ruskin University, Cambridge, UK; i Centre for Health, Performance and Wellbeing, Anglia Ruskin University, Cambridge, UK

**Keywords:** Athletes, physical activity, long-COVID, COVID-19, rehabilitation, return to play, recommendations

## Abstract

Currently, there is limited research reporting the symptoms of long COVID among athletes, and the recommendations for athletes returning to competition/training who have experienced long COVID symptoms. Therefore, the aim of this systematic review is to synthesise the recommendations for returning athletes who have experienced long COVID symptoms. The protocol was registered in PROSPERO under CRD42021265939. Two authors searched the electronic databases PubMed, Embase, Scopus, the Cochrane Library, Web of Science, CINAHL, PsycINFO, and SPORTDiscus from August 2019-July 2021. Search terms included words related to “long COVID”, “athlete” and “return”. Data extraction was completed for each study by two independent investigators for: (1) first author name; (2) year of publication; (3) journal; (4) Definition of athlete (i.e. elite or non-elite) (5) Recommendations reported. A total of 220 records were found. Following title and abstract screening, 61 studies were eligible for full text screening. Overall, no studies, commentaries, editorials or reviews provided specific recommendations for “long COVID” defined as COVID-19 signs and symptoms lasting for over 4 weeks as a result of COVID-19 infection. In addition, we found no studies which reported symptoms of athletes suffering from long COVID. Despite the lack of evidence, we did find eight separate professional recommendations for managing “long-term effects” and “ongoing” or “prolonged” symptoms and COVID-19 complications among athletes. Practitioners should be aware of both mental and physical symptoms of long COVID, and additional considerations may be required for athletes who have undergone intensive care. The present review provides a list of recommendations based on existing literature that may be followed and implemented for returning athletes.

**Key Messages**
- Further research, including longitudinal research of athletes who have tested positive for COVID-19, is required to develop evidenced-based guidelines for athletes with ongoing COVID-19 symptoms.
- Prior to returning to play after COVID-19 infection, a thorough medical history, physical and psychological examination should be conducted by a medical professional.
- Athletes should continue to monitor and record their own physical and psychological markers of health.

## Introduction

The unprecedented rate at which COVID-19 has spread worldwide, as well as its scale of reach and the prolonged duration of the pandemic, was unexpected. This new, highly contagious disease moved rapidly across China and spread to more than 200 countries, infecting 233,503,524 people by October 2021 [1]. COVID-19 signs and symptoms can be classified in three stages. The first stage of COVID-19 infection is defined as ‘Acute COVID-19′ and refers to the initial signs and symptoms of COVID-19 which last for up to 4 weeks. ‘Ongoing symptomatic COVID-19′ is defined as signs and symptoms lasting 4–12 weeks, whilst “post-COVID-19 syndrome” is defined as signs and symptoms of COVID-19 infection that last longer than 12 weeks attributable to COVID-19 infection. “Long COVID” is used to describe “signs and symptoms that continue or develop after acute COVID-19, it includes both ongoing symptomatic COVID-19 and post‑COVID-19 syndrome” [2]. The most commonly reported symptoms of long COVID are: fatigue, breathlessness, muscle soreness, chest pain, difficulty concentrating, anxiety, and depression [3]. To date, the vast majority of studies have focussed on acute COVID-19 with few focussing on long COVID [4].

However, recent studies have suggested that the prevalence of long COVID is high. For instance, according to WHO data, around 25% of people who have had COVID-19 experience at least four weeks of symptoms, and 10% of people who have had COVID-19 experience symptoms for more than 12 weeks [5]. Moreover, a UK-based study estimated 945,000 people living in private households were experiencing long COVID symptoms in July 2021 [6]. The symptoms of long COVID can be debilitating, with 19.3% of those with self-reported long COVID reporting that their ability to carry out normal activities of daily living had been limited “a lot” [6].

A review of long COVID research found evidence that the term “long COVID” includes three previously identified syndromes including: post-intensive care unit syndrome (PICS), long-term organ damage, and post-viral syndrome [7]. In addition, long COVID may also be an active disease, characterised by an inflammatory response, continued viral activity and a risk of blood clotting disorders [7].

Exercise has been recommended to reduce the risk of severe infection in the general population, and for patients recovering from COVID-19, to reduce the impact of physical de-conditioning associated with the disease. Moreover, exercise may be particularly beneficial to aid in the recovery of the musculoskeletal system following COVID-19 infection. Indeed, a recent review concluded that from the onset of COVID-19 symptoms and through the most severe stages of the disease, musculoskeletal symptoms, including myalgia, arthralgia, and fatigue, were frequently experienced [8]. Considering this, specific considerations are required for athletes who are more likely to engage in strenuous exercise regimes than the general population, which may increase risk of further COVID-19 complications [9].

An important goal for sporting organisations and associated staff (e.g. managers, coaches, sport physicians etc.) is to supervise athletes in returning to training and competition following an illness or injury. This is of upmost importance for the professional athlete as uninterrupted training often at a high intensity is essential for both athletic and career progression. Therefore, in the context of long COVID decisions relating to “return to play” need to balance the potential adverse impact of training on health while experiencing symptoms of long COVID with the diminishing levels of fitness, from a pause in training or training at a low intensity, that will impact sporting performance [10].

However, currently there is limited research which reports the symptoms of long COVID among athletes, and the recommendations for athletes returning to competition/training who have experienced long COVID symptoms. Therefore, the aim of this review is to synthesise the recommendations for returning athletes who have experienced long COVID symptoms.

## Method

The protocol was registered in PROSPERO under CRD42021265939. Two authors searched the electronic databases PubMed, Embase, the Cochrane Library, Web of Science, CINAHL, PsycINFO, and SPORTDiscus from August 2019-July 2021. Search terms included words related to “long COVID”, “athlete” and “return-to-play” (Appendix 1). In addition, we hand searched the reference lists of eligible articles.

All study designs were eligible providing they met the following inclusion criteria:

1. Studies that reported recommendations for athletes (elite and non-elite) who have suffered from long COVID-19 symptoms (defined as over 4 weeks or more of COVID-19 symptoms).
2. Studies that reported symptoms of athletes suffering from long COVID.
3. Studies in English, Spanish, French, or Italian.
4. Reviews, expert consensus, professional viewpoints and editorials which included specific recommendations.
5. Peer reviewed articles.

Studies outside the language criteria were excluded.

Title, abstract, and full-text screening was performed in pairs and any disagreement between reviewers was resolved by a third senior reviewer.

### Data extraction

Data extraction was completed for each study by two independent researchers for: (1) first author name; (2) year of publication; (3) journal; (4) definition of athlete (i.e. elite or non-elite) (5) recommendations which applied to athletes with ongoing Covid-19 symptoms. As we did not find any studies which reported data from athlete populations with long COVID, we did not extract data on the symptoms, length of COVID symptoms, type of sport, study design and population demographics as planned in our protocol.

Following data extraction we synthesised the literature to provide an overview of the recommendations for each syndrome which can be classified as “long COVID” [7], including:

1. Post-intensive care unit syndrome (PICS): cognitive, psychiatric, and/or physical disability after treatment in an intensive care unit [11].
2. Long-term organ damage: damage to the organs which persists after Covid-19 infection.
3. Post-viral syndrome: persistent symptoms at the post-viral stage [12].
4. Long COVID as a distinct syndrome and active disease characterised by an inflammatory response, continued viral activity and a risk of blood clotting disorders [7].

## Results

**Figure 1.**
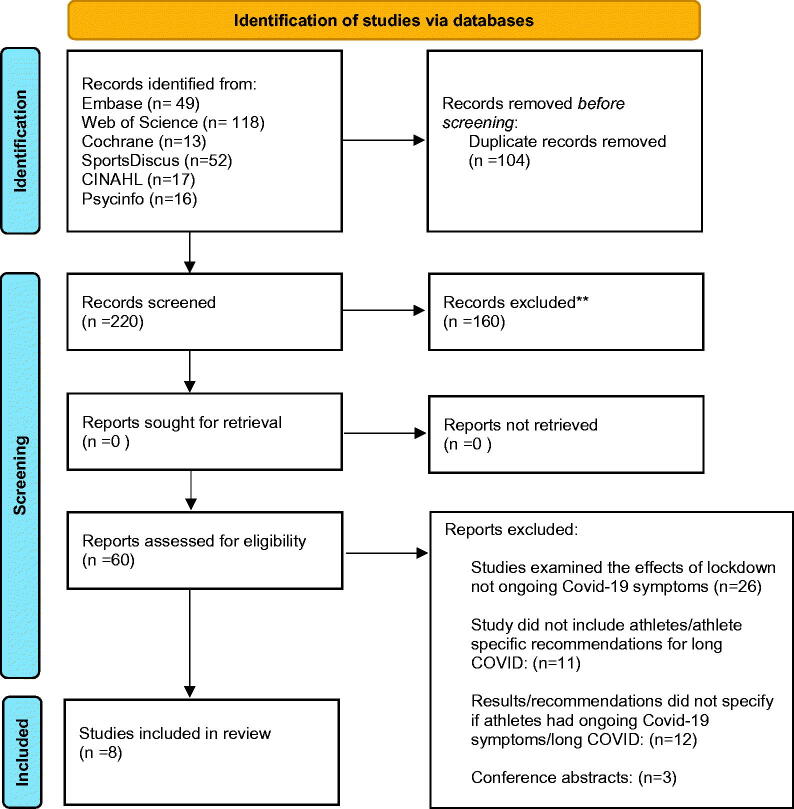
PRISMA flow diagram. From ref. Page et al. [30]. For more information, visit: http://www.prisma-statement.org/

A total of 220 records were found. Following title and abstract screening 60 studies were eligible for full text screening (Figure 1). Overall, no studies, commentaries, editorials or reviews provided specific recommendations for “long COVID” defined as COVID-19 signs and symptoms lasting for over four weeks as a result of COVID-19 infection. In addition, we found no studies which reported symptoms of athletes suffering from long COVID. However, we did find eight separate professional recommendations for managing “long-term effects” and “ongoing” or “prolonged” symptoms and COVID-19 complications among athletes (Table 1). Due to the lack of experimental or observational studies, we did not use the Newcastle Ottawa Scale to assess the study quality as planned in the protocol.

**Table 1.** Recommendations for athletes with ongoing COVID -19 symptoms.

Author and year	Title	Athlete definition	Recommendations
Kim et al. 2021 [13]	Coronavirus Disease 2019 and the Athletic Heart	Athletes involved in competitive sport	Cardiovascular testing should be considered on an individualised basis for athletes with protracted symptoms (≥10 days). Testing should include a clinical evaluation, ECG, hs-cTn (or available cTn) level test, and echocardiography.
	Emerging Perspectives on Pathology, Risks, and Return to Play.		Further testing may include CMR, exercise testing, and extended-duration ambulatory rhythm monitoring, if symptoms persist or recur.
Halle et al. 2021 [14]	Exercise and sports after COVID-19-Guidance from a clinical perspective	Individuals who engage in leisure time or competition level sport/exercise.	A thorough clinical examination should be taken to identify prolonged organ impairment.
			Recommended tests for athletes include: resting ECG for all people recovering from Covid-19 and returning to physical activity, including an echocardiography for people with ongoing mild symptoms. Cardiac MRI may be needed if tests are suggestive of myocarditis.
			An exercise ECG including measures of oxygen saturation is advisable, including lactate testing including oxygen saturation measurements during exercise or blood gas analysis before and after maximal exercise is advisable among people with persistent respiratory limitations. People with post‐pneumonia or myocarditis should undergo spiroergometry with blood gas analysis.
			Maximal CPET or exercise protocol including lactate testing can guide prescribed training intensity when an athlete returns-to-play.
			Measurements of residual volume and total lung capacity by body plethysmography recommended for athletes with signs of persistent respiratory limitations, alongside static and dynamic lung function testing.
			Measurement of inspiratory muscle strength capacity may be considered among athletes with ongoing dyspnoea or unexplained exercise limitation.
			A clinical neurological examination including motor, sensory, and coordination testing, cranial and peripheral nerves should be taken.
			Re-examination including resting ECG and blood tests should be performed at 3–6 months to assess any long-term effects which may occur.
Schellhorn et al. 2020 [15]	Return to sports after COVID-19 infection	Not specified.	The development of cardiovascular complications and long-term consequences of COVID-19 need to be ruled out by follow up (physical exam, resting and exercise ECG, and echocardiography).
Wilson et al. 2020 [16]	Cardiorespiratory considerations for return-to-play in elite athletes after COVID-19 infection: a practical guide for sport and exercise medicine physicians	Elite athletes.	Athletes with persistent symptoms (longer than 14 days) should undergo a thorough history and physical examinations including:
			Cardiac examination: 12-lead ECG and Cardiac MRI
			Respiratory examination: Chest X-ray and lung function
			Biochemistry evaluation: High-sensitivity cardiac troponin T, C-reactive protein and D-Dimer.
			The psychological impact of prolonged recovery among athletes should also be considered.
Elliott et al. 2020 [17]	Infographic. Graduated return to play guidance following COVID-19 infection	Performance athletes who have mild-moderate illness.	Athletes with complicated or prolonged COVID-19 may need further examination including: Blood tests (high sensitivity-Troponin, Brain Natriuretic Peptide and C reactive protein), cardiac monitoring (12-lead ECG, echocardiogram, exercise tolerance test and cardiac MRI), respiratory (spiromtry), renal and haematological monitoring.
Verwoert et al. 2020 [18]	Return to sports after COVID-19: a position paper from the Dutch Sports Cardiology Section of the Netherlands Society of Cardiology	All athletes aged 16 and over.	A record of COVID-19 cases among athletes and highly active individuals, including follow-up, is needed to inform future return to sport guidelines.
McKinney et al. 2020 [19]	COVID-19–Myocarditis and Return to Play: Reflections and Recommendations From a Canadian Working Group	Highly active persons who exercise or compete regularly at either a recreational or a competitive level.	If new/ongoing cardiac symptoms are present (palpitations, syncope, chest pain, dyspnoea, unexplained increase in heart rate), athletes should not engage in moderate-high intensity exercise, and undergo cardiac history assessment and physical examination.
Biswas et al. 2021 [20]	The BASES (British Association of Sport and Exercise Sciences) Expert Statement on Graduated Return to Play Following Covid-19 infection.	Elite and sub-elite athletes	Due to the risk of longer-term complications, return to play should be overseen or at least signed off by a medical doctor.

**Figure 2.**
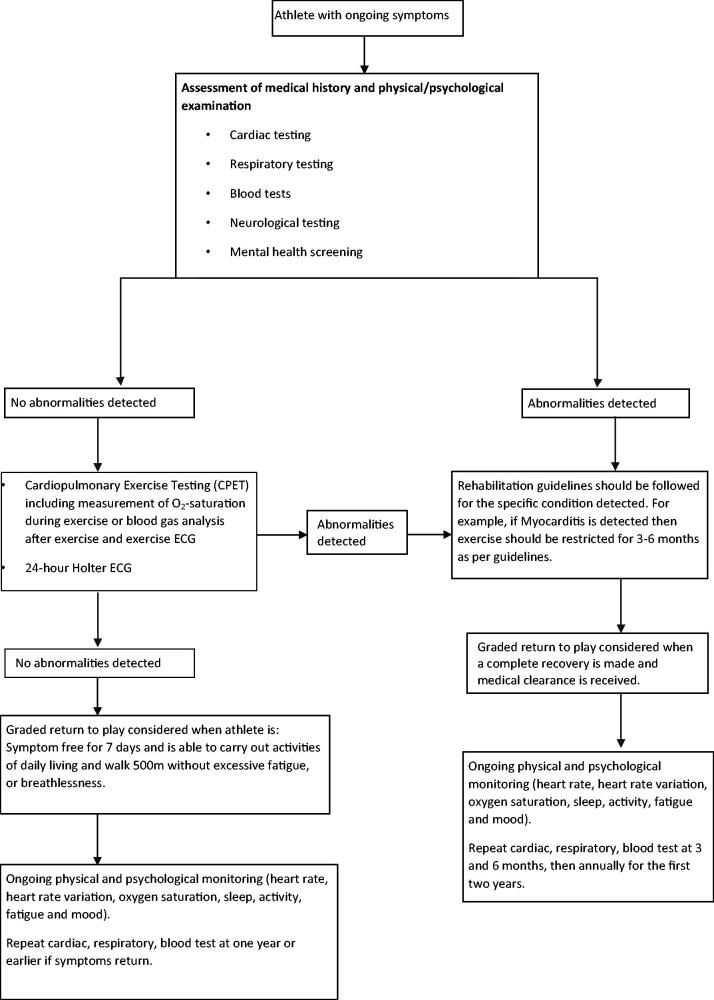
Flow diagram of athlete protocol for managing ongoing COVID-19 signs and symptoms.

Figure 2 is based on the recommendations across the literature, for athletes returning to exercise following ongoing COVID-19 symptoms.

## Discussion

Given that long COVID has been used as an umbrella term to refer to four syndromes: PICS, long-term organ damage, post-viral syndrome and an active disease, we will discuss the recommendations made for each syndrome in turn, and then provide an overall summary of the recommendations for the management of long COVID.

### Post-intensive care unit syndrome (PICS)

Research has shown that athletes are at lower risk of severe COVID-19 symptoms than the general population. However, for people who have been admitted to an intensive care unit, including athletes, there may be a risk of PICS. PICS refers to a combination of emotional and physical symptoms following intensive care [21]. No studies reported the symptoms of PICS among athletes, or provided recommendations for athletes experiencing PICS. However, the BASES consensus on return to play recommends that anyone who required hospitalisation due to COVID-19 infection should be managed by a multi-disciplinary team including medical specialists [17].

### Long-term organ damage

Research has found COVID-19 may manifest in various organs including the heart, lungs, kidney, intestine and the brain [22]. Several studies referred to the risk of long-term organ damage among athletes, in particular the risk of myocarditis and pneumonia, which are associated with COVID-19 infection. The literature suggested an array of tests to assess the physical health of athletes that will likely identify any long-term organ damage. It should also be noted here that among these tests are also assessments to monitor mental health and thus will be discussed here also.

Table 2 presents the rationale for different tests recommended in the literature, which may be selectively done according to the athletes signs and symptoms.

**Table 2.** Tests to assess physical (and mental) health of athletes.

Measures taken	Reason for taking the measure
Cardiovascular tests	
1) 12-lead ECG, echocardiography, cardiac MRI^a^	Screen for cardiovascular complications, identify Myocarditis/myocardial oedema
Cardiopulmonary tests	
1) CPET with ECG including measurement of respiratory efficiency, O2-saturation during exercise or blood gas analysis after exercise (Recommended by Halle et al. for people with more severe symptoms and/or post‐pneumonia or myocarditis).	Identify subclinical impairment in cardiopulmonary system, e.g. arrhythmias, premature dyspnoea, impaired diffusion capacity and guide future training intensities if returning to play.
Respiratory tests	
1) Chest X-ray	Identify Pneumonia
Spirometry: Bronchodilator challenge and bronchoprovocation testing	Identify airway disease (may have been undiagnosed or exacerbated due to COVID-19)
Spirometry: Vital capacity and forced expiratory volume	Identify impaired lung function
FeNO testing	Identify post inflammatory bronchoconstriction
2) CT Thorax (if significant suspicion of thromboembolic or intrapulmonary abnormalities)	Covid-19 related lung damage and pulmonary vasculature pathology
3) Ventilation–perfusion scan (if O2 desaturation during CPET testing is identified and CT thorax scan is normal)	Identify microemboli.
4) Body plethysmography (Recommended by Halle et al. for people with identified pulmonary problems or ongoing symptoms of pulmonary disfunction e.g. breathlessness).	Measure residual volume and total lung capacity
5) Inspiratory muscle strength assessed	To identify potential causes behind unexplained exercise limitation/dyspnoea
Blood tests	
1) D-dimer	Identify pulmonary embolism
Troponin (hs-cTn or cTn levels test)^b^	Detect damage to the heart
Brain Natriuretic Peptide	Detect heart failure
C reactive protein	Assess inflammation
Blood cell count	Assess inflammation
Creatinine	Assess kidney function
Neurological test	
1) Clinical neurological exam	Test for signs of damage to the neurological system, and to test for risks to safety during exercise e.g. symptoms of Vertigo.
Musculoskeletal test	
1) Musculoskeletal assessment, e.g. Strength, flexibility, muscular endurance.	Musculoskeletal assessment may be advisable to identify areas of weakness following a period of deconditioning.
Mental health	
1) Injury-Psychological Readiness to Return to Sport	To help identify if referral to mental health specialist/sports psychologist may be beneficial.
Mental health screening i.e. screen for symptoms of depression, anxiety.	To help identify if referral to mental health specialist/sports psychologist may be beneficial.

CPET: Cardiopulmonary exercise testing; ECG: Electrocardiogram.

^a^Athletes may have anomalies in cardiac screening as a result of sport/exercise training, e.g. wall motion anomalies and pericardial effusion [13].

^b^Raised troponin levels may also be an outcome of high intensity sport/exercise^13^.

All athletes are recommended to undertake a medical history and physical examination. For athletes with persistent symptoms, it is recommended that a 12-lead ECG and MRI are conducted to identify myocarditis and/or myocardial oedema. If myocarditis or myocardial oedema is detected, then rehabilitation guidelines should be followed specific to these complications. Following a cardiac assessment, several studies suggest performing cardiopulmonary exercise testing (CPET).

Respiratory tests are also recommended following ongoing symptoms, including chest X-ray, conducting spirometry and Exhaled Nitric Oxide testing (FeNO testing). Following any abnormalities in readings or persistent unexplained symptoms, the following measures may provide a more comprehensive assessment: CT Thorax, ventilation–perfusion scanning, body plethysmography and testing inspiratory muscle strength.

Blood tests are also a useful tool to assess for biomarkers of ongoing damage to the organs. Blood tests referenced in the literature included: D-dimer, Troponin, Brain Natriuretic Peptide, C-reactive protein, blood cell count and Creatinine, to identify possible organ damage. We also suggest that assessing muscle enzymes (Creatine Kinase and adolase) may be useful, as previous studies have reported an association between Covid-19 and rhabomyolysis. Even though rhabomyolysis is resolved after Covid-19, exercise can aggravate muscle injury [23].

In addition to cardiac, pulmonary and blood tests, Halle and colleagues also recommend clinical neurological and musculoskeletal testing [14]. We suggest that for athletes who have undergone a baseline assessment of concussion symptoms using the sports concussion assessment tool (SCAT) [24] prior to COVID-19 contraction, it may be of value to compare symptom evaluation, cognitive screening, neurological screening and delayed recall values recorded in a SCAT test to post-COVID-19 values to identify significant differences pre and post scores which may warrant further evaluation. In terms of musculoskeletal testing, testing should be carried out to identify any areas of pain or weakness, which may impede an athlete’s return to play. A study found that muscle pain was common among those hospitalised with COVID-19 [25]. In addition, a period of de-training due to COVID-19 illness may have also resulted in muscle weakness or tightness which could contribute to future injury risk. Muscle pain, weakness or tightness may need to be addressed by a physiotherapist or a strength and conditioning coach.

Mental health screening was not included in the primary evaluation of athletes returning to play. However, given that population studies have found symptoms of anxiety and depression among people suffering from long COVID, we have included mental health screening in the initial evaluation. Examples of mental health screening tools which may be of value are the Injury- Psychological Readiness to Return to Sport Scale (I-PRRS) recommended by BASES as a monitoring tool during a graded return to play [20], and screening for depression and anxiety. However, ultimately mental health evaluation should be tailored to the needs of the athletes. For example, athletes may feel more comfortable discussing their mental health with a sports psychologist than using a screening tool presented by a doctor. Athletes with previous history of mental health problems should also be offered tailored support e.g. an athlete with an eating disorder may need to seek specialist support.

### Post viral syndrome

A review of long-term Covid symptoms found the most commonly reported long-term outcome of COVID-19 was fatigue [26]. The BASES consensus on return to play following COVID-19 infection recommends that athletes should be able to complete activities of daily living and walk 500 m without excessive breathlessness or fatigue. If the athlete can carry out the activities of daily living without excessive fatigue and has been symptom free for 7 days with no further structural damage from COVID-19, athletes are recommended to follow a medically supervised graded return to play. It should be noted that the athlete should not be undergoing any treatment or medication which could mask COVID-19 symptoms prior to being cleared to return to play. BASES also suggests 2–3 days graded return to play for everyday lost to illness. When return to play is resumed, then training should be increased firstly by frequency, then duration and finally intensity [20]. The Stanford Hall consensus statement for post-COVID rehabilitation recommends that the first week of returning to exercise should consist of low level stretching and light muscle strengthening activity [27]. In addition, ongoing monitoring including physical and psychological measures, for example, heart rate, heart rate variation, oxygen saturation, sleep, activity, fatigue and mood should be continued to ensure that return to play is graded, and that rest and recovery is sufficient. In female athletes, monitoring the menstrual cycle may also be beneficial to identify if psychological and biological fluctuations may be explained by changes in fluctuating hormones. Menstrual cycle monitoring can also be an indication of overall health e.g. stress management and energy balance [28].

Interestingly, the National Institute for Health Research draws parallels between the symptoms and causes of long COVID symptoms and overtraining syndrome (OTS) [7]. OTS is a more extensively researched syndrome in the field of sports science than long COVID, and in light of a lack of specific guidance regarding return to play following long COVID, drawing upon guidance recommended for OTS may assist practitioners and athletes with the management of long COVID fatigue. For example, it is recommended to manage OTS, that athletes and practitioners maintain regular competition and training records of performance, avoid excessively monotonous training, are aware of external stresses and how to minimise their impact, and that regular athlete-practitioner communication is maintained [29]. It is also important that practitioners do not misdiagnose long COVID as OTS.

### Long COVID as a distinct disease

Finally, long COVID has also been identified in recent studies as potentially an active disease, with an inflammatory response and viral activity, and occasionally blood clotting disorders. As previously discussed, the literature recommends that return to play does not resume until the athlete has rested for 10 days, is 7 days symptom-free without treatment or medication for symptoms and does not have any structural organ damage. Therefore, if an athlete is experiencing active COVID-19 disease then they should not return to play. It is important to highlight the possibility of ongoing active COVID-19 disease, as athletes with ongoing viral and inflammatory activity may be at heightened risk of structural organ damage. Thus, reinforcing the need for repeated testing for organ damage among athletes who are experiencing ongoing symptoms.

### Limitations and recommendations

Our review was largely limited due to the ambiguity of the term “long COVID” in the literature. Long COVID is a relatively recent identified condition, and future studies which examine long COVID should consider that different pathologies may result in similar symptoms. In addition, whilst there is recognition that athletes who are recovering from or have COVID-19 may be at risk of mental health problems, there were limited recommendations for how symptoms of poor mental health may present in the long-term and also how to minimise these symptoms. To add to this, recommendations were not stratified by sport, or by athlete level of play i.e. elite vs recreational athlete, instead recommendations were often stratified by “mild/moderate symptoms” and “severe symptoms”. However, long-term COVID-19 symptoms can occur in those who experience mild/moderate and severe symptoms of COVID-19. Research which explicitly studies the long-term effects of COVID-19 among athletes and provides evidence-based recommendations for athletes with long-term symptoms is needed. Particularly as many of the recommendations for athletes require intensive medical investigation. Given the lack of evidence for the effects of long COVID for athletes, it may be assumed that many of these test are precautionary measures, which could be costly and potentially unethical to conduct if evidence emerges of a limited long term clinical impact of Covid-19 among athletes. Sport-specific, and level of athlete recommendations may also be warranted. For example, it is plausible a recreational athlete may have limited resources to continue home monitoring (e.g. heart rate monitoring using ECGs) and to carry out the range of tests required to confirm return to play is safe, therefore, feasible guidelines may be needed for recreational athletes. Different levels of physical fitness between elite and recreational athletes may also be important when developing specific recommendations due to different physical fitness and physical capacity levels.

## Conclusion

Current literature on long COVID has highlighted a range of syndromes which may be defined as long COVID. Practitioners should be aware of both mental and physical symptoms of long COVID, and additional considerations may be required for athletes who have undergone intensive care. Overall, based on the current literature, it is suggested that an athlete seeks medical clearance prior to returning to training or competition. A multidisciplinary medical clearance should include a comprehensive screening of the cardiovascular, pulmonary, neurological and muscular systems. Appropriate and tailored services, including tele-health support, should be available to athletes during the infection in order to closely follow-up the disease course. If there is no structural damage and no further symptoms of COVID-19 for at least 7 days, then a graded return to play may be resumed. Continuous daily monitoring can be undertaken by the athlete, being recorded and reported to practitioners and coaches. Clinical re-evaluation should be undertaken a maximum of one year later, although it may be beneficial to carry out re-evaluation earlier if the athlete is considered at risk. If symptoms return, it may be an indication that the athlete is experiencing continued inflammation and viral load as a result of long COVID. In this case, we recommend a suspension of training/competition followed by immediate re-evaluation of symptoms and organ damage status. Importantly, the current recommendations for the management of long COVID among athletes should continue to be adapted in light of new high-quality evidence, including longitudinal research.

## Data Availability

On reasonable request to the corresponding author.

## Appendix 1

((LONG OR LONG-TERM OR post-acute OR long-tail OR persistent OR ‘long term’) AND (CORONAVIRUS* OR COVID-19 OR COVID OR SARS-CoV-2)) OR (‘post COVID syndrome’ OR ‘post-covid syndrome’ OR ‘long haul covid’ OR ‘covid long haulers’))) AND (ATHLE* OR SPORT* OR ‘athlet* rehab*’ OR ‘pro* athlete*’ OR ‘sport* rehab*’ OR ‘return to play’ OR ‘return to train*’ OR ‘athle* recovery’ OR ‘sport* recovery’ ‘return to competition’ OR ‘competition return’).
